# Addressing data gaps in sustainability reporting: A benchmark dataset for greenhouse gas emission extraction

**DOI:** 10.1038/s41597-025-05664-8

**Published:** 2025-08-27

**Authors:** Jacob Beck, Anna Steinberg, Andreas Dimmelmeier, Laia Domenech Burin, Emily Kormanyos, Maurice Fehr, Malte Schierholz

**Affiliations:** 1 LMU Munich, Department of Statistics, Munich, 80539 Germany; 2 Munich Center for Machine Learning, Munich, 80538 Germany; 3 Deutsche Bundesbank, Research Data and Service Centre, Frankfurt am Main, 60431 Germany

**Keywords:** Attribution, Economics

## Abstract

Reliable company-level greenhouse gas (GHG) emissions data are essential for stakeholders addressing the climate crisis. However, existing datasets are often fragmented, inconsistent, and lack transparent methodologies, making it difficult to obtain reliable emissions data. To address this challenge, we present a gold standard dataset containing emission metrics extracted from 139 sustainability reports collected from company websites. This dataset acts as an intermediate step to validate and fine-tune models for large-scale extraction of emissions data from thousands of reports. We employ a Large Language Model (LLM)-powered extraction pipeline to automatically extract emissions metrics. These values are then independently assessed by two non-expert annotators. Reports with full agreement are directly considered gold standard, while discrepancies undergo expert review in two stages, with remaining disagreements resolved through in-person discussions. This structured process ensures high data quality while reducing reliance on experts. Our dataset serves as a benchmark for human and automated annotation, with significant reuse potential for information extraction tasks in sustainable finance as well as other downstream tasks such as greenwashing analysis.

## Background & Summary

Company-level greenhouse gas (GHG) emissions data are critical for a wide range of stakeholders, including private and public actors, due to their importance in addressing the climate crisis. Against the backdrop of an increasing adoption of carbon pricing schemes around the world^1^, policymakers require accurate emissions data to assess the impacts and effectiveness of these policies. Similarly, the financial sector needs consistent and comparable data to regulate climate-related risks and to assess the exposure of banks and financial institutions to emissions-intensive companies^2^. Furthermore, analyses of emissions data support the development of climate litigation, aimed at holding entities accountable for historical responsibilities^3^, and are essential for crafting financial instruments that integrate climate considerations^2^. For the general public, these data enable informed debates, facilitate accountability, and guide consumption choices. Despite their critical importance, obtaining reliable company-attributed emissions data remains an unresolved challenge. Current datasets, provided by a mix of non-profit and commercial vendors, are often inconsistent^4^ and reliant on non-transparent methodologies and non-standardized reporting^5^. This lack of standardization undermines efforts to ensure the comparability and accuracy of emissions data.

**Fig. 1:**
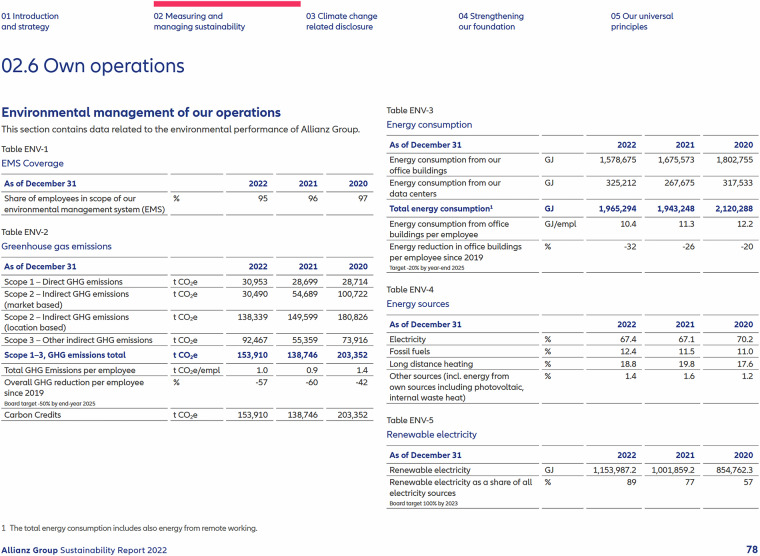
Example emission table from Allianz (2022, p.78).

**Fig. 2:**
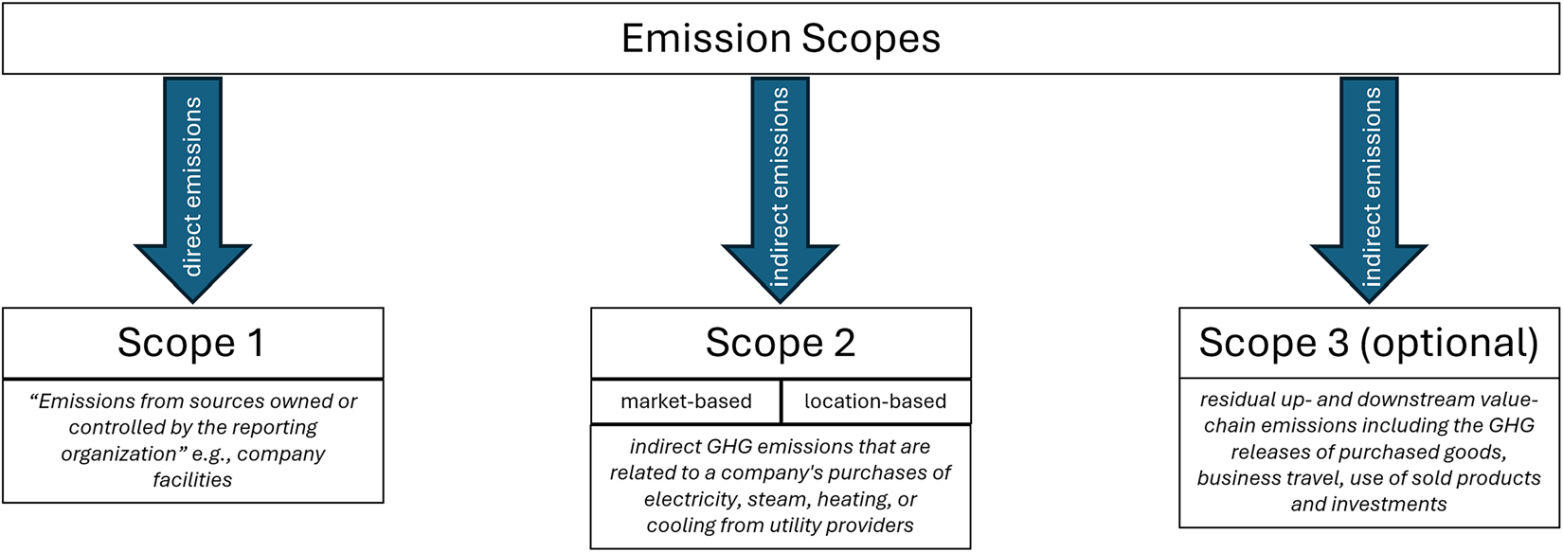
Visual illustration of GHGP guidance. Based on World Resource Institute (WRI) and World Business Council for Sustainable Development (WBCSD), 2011.

The accounting of GHG inventories first emerged in the 1990s in the context of voluntary corporate social responsibility reporting^6,7^. In recent years, GHG accounting has become mandatory for companies in various jurisdictions. For example, in the EU, large companies (i.e., companies with more than 500 employees, balance sheet total exceeding 20 million €, or net turnover exceeding 40 million €) are required to report their GHG emissions as well as other climate-related metrics, according to the Non-Financial Reporting Directive (NFRD). This directive from 2014 has been replaced by the more comprehensive Corporate Sustainability Reporting Directive (CSRD) (Directive 2022/2464) in 2023, but is currently subject to review and is yet to be implemented by multiple EU member states. In addition, emission disclosures have been integrated into the international financial reporting standards (IFRS). GHG accounting results are usually communicated through a variety of non-standardized and unstructured PDF reports, often consisting of 100 pages or more, which are uploaded to company websites rather than a central repository (see Fig. 1 for an example). While these reporting frameworks and standards vary in their definitions and data requirements, Jia *et al*. 2022 [^8^, p.13–18] find that they all reference the GHG accounting developed by the Greenhouse Gas Protocol (GHGP) or, alternatively, the ISO 14000 standards, which can be harmonized with the GHGP categories^9^. The GHGP framework offers guidance to companies on how to define their organizational boundaries and how to define and segment the operational boundaries of the emissions that they account for (see Fig. 2). As to the operational boundaries, the GHGP differentiates between three Scopes, reflecting varying degrees of “ownership”. The unit for all the disclosed emissions is *CO*_2_ equivalents (*CO*_2_*e*), which converts different GHG into a single metric based on the Global Warming potential as reported by the Intergovernmental Panel on Climate Change (IPCC). Scope 1 emissions are defined as “Direct GHG emissions (...) from sources that are owned or controlled by the company”^9^(p.27) and include GHG releases from manufacturing processes, company vehicles, and buildings. Scope 2 covers indirect GHG emissions that are related to a company’s purchases of electricity, steam, heating, or cooling from utility providers. Since 2015, Scope 2 emissions can be disclosed according to two different approaches; location-based and market-based methods. Market-based disclosures represent emissions tied to a company’s contracted electricity providers, while the location-based method reflects the average emissions’ intensity of grids on which energy consumption occurs. According to the GHGP supplemental guidance^10^ companies should disclose Scope 2 emissions according to both methods, unless they lack data for the market-based method, in which case only location-based emissions should be reported. Finally, Scope 3 emissions cover 15 categories of residual up- and downstream value-chain emissions, including the GHG releases of purchased goods, business travel, use of sold products, and investments^11^. Unlike Scope 1 and 2 emissions the reporting of Scope 3 is optional according to the GHG protocol^9^.

The GHGP has been criticized for allowing companies multiple options on how to calculate and consolidate their emission inventories, thus giving rise to inconsistencies^8,12^. These complications regarding the determination of companies’ emissions are also reflected in existing approaches to generate emission datasets. While there are several non-profit and commercial data vendors of corporate GHG emissions, the values that they provide for the same company-year-scope combinations do not always match. In a comparison among seven providers, Busch *et al*.^4^ find pairwise correlations to be generally high (0.93–0.99) for Scope 1 emissions, while there is less agreement for Scope 2 and 3 values, with the latter category displaying correlations as low as 0.22. The lack of consistency and transparency within and between existing datasets underlines a significant data gap that makes it difficult to assess and compare GHG emissions across different companies, years, and scopes. The problems related to the aggregation of reported emissions also have implications for training and evaluating automated information extraction approaches, as researchers have had to develop ad hoc annotation strategies tailored to their specific use cases^13^.

**Fig. 3:**
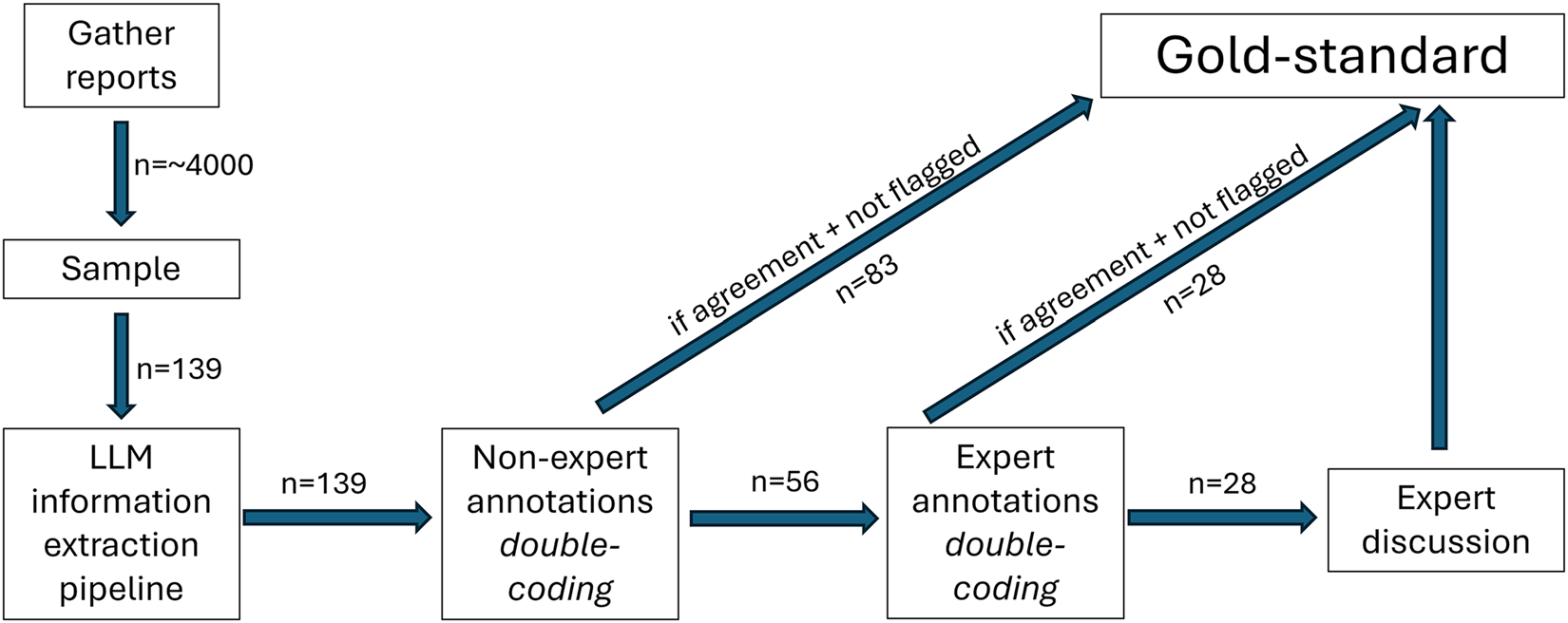
Data collection overview graphic.

We intend to address this data gap by providing a gold standard dataset that contains the scope emission metrics stated in 139 sustainability reports while ensuring a satisfactory degree of comparability between reports. To achieve this, we put high effort in a sound and unambiguous annotation convention and deploy domain expert capacities extensively to prioritize annotation accuracy over quantity. Additionally, we demonstrate the potential of leveraging Large Language Models (LLMs) and non-experts in expert domain annotation, to reduce the workload of domain experts. Our data structure facilitates a quality assessment of LLM- and non-expert annotators, allowing us to determine when the involvement of human domain experts is needed and when it is not. In that, our data serves as a benchmark for other human or automated annotation/information extraction setups and inherits large reuse value due to the gold standard nature of the emission metrics. For an overview of the data collection process see Fig. 3, for a detailed description we refer to the Methods section.

The remainder of the paper is structured as follows: The methods section chronologically describes the sequential data collection and annotation steps. Subsequently, we illustrate data structure and provide descriptive statistics, followed by a presentation of the data quality checks. Ultimately, we give instructions for the reuse of our data and outline the setup of our data and code repositories.

## Methods

### Overview

The data collection process follows the structure illustrated in Fig. 3. Given a sample of 139 sustainability reports available on the companies’ websites (URLs to retrieve the reports are provided on Zenodo), we utilize a data extraction pipeline based on an LLM to automatically extract emission metrics. Subsequently, two human non-expert annotators independently assess and correct the LLM-extracted emission values. In case of agreement for all entries of a report, the report is immediately considered as gold standard. In case of disagreement between non-experts a report is routed to two pairs of expert annotators. Cases where the two groups agree are again transferred to the gold standard dataset. Both, the non-experts and the experts have the option to flag unclear records for additional review. The remaining disagreements between the two expert annotator groups are resolved in an in-person expert discussion and the resulting entries transferred to the gold standard dataset. We describe this process in detail in the subsequent paragraphs.

### Sustainability Report Collection

Due to differences in regulations across jurisdiction regarding the obligations, format and scope of affected companies, there is some uncertainty about the total population of sustainability reports. Collating information from different financial data providers, the OECD has estimated that of the 43970 listed companies globally, about 9600 disclosed sustainability-related information in 2022 or 2023. Out of these, 6308 companies disclosed Scope 1 and Scope 2 emissions, while 4246 disclosed Scope 3 emissions in the analysed period [^14^, p.13–15]. For this gold standard dataset we consider a curated repository of publicly available reports, collected and maintained by Deutsche Bundesbank data scientists. This repository contains sustainability reports and annual reports of a list of around 4000 companies from 2017 to 2022. The longitudinal nature allows exploring whether companies have changed their adherence to the GHGP in this time span. The featured companies are the ones included in two major indices: the international “MSCI World Small Cap” and the German “DAX”. As a result the companies represent a diverse group varying in sectors and regions with a potentially higher representation of smaller companies due to the nature of the MSCI Small Caps index.

We then draw a stratified sample of 139 reports, published by 132 companies, such that the sample distribution of the companies’ first letters matches the full set of sustainability reports. Companies, irrespective of size or industry, are drawn at random. This sampling design choice is made to create a sample that includes smaller companies and those from diverse backgrounds, to provide insights of the various challenges one might face during annotation.

After this initial step, we conduct a cursory expert review and exclude 36 reports from the sample. The reasons for the exclusion of these documents were that they were either incomplete (e.g., only containing the first pages of a longer document) or did not correspond to our definition of an eligible report. We deem annual reports as well as separate, stand-alone sustainability, Environment, Social & Governance (ESG) or Corporate Social Responsibility (CSR) reports as eligible reports for our dataset, collectively referring to all these types as *sustainability reports* throughout this publication. Consequently, documents such as investor presentations, news releases, half-yearly reports and questionnaire response sheets were excluded. Due to this, 36 reports were excluded. To augment our reduced sample, we substitute excluded reports with 36 eligible reports from the repository, following the same sampling strategy as described above.

### LLM Extractions

**Fig. 4:**
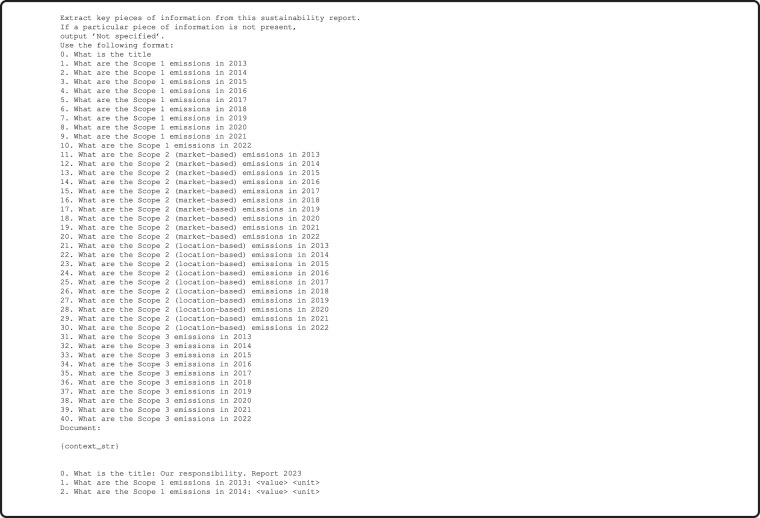
Prompt passed to GPT-4 to extract emissions for three scopes and years 2013–2022. For {context_str} we insert the different page texts.

To extract the initial set of emissions metrics from the reports, we reuse the Retrieval Augmented Generation (RAG)-inspired pipeline developed by Dimmelmeier *et al*.^15^. First, the sustainability reports are loaded page-wise to a vector database. Then we search for the most relevant pages with respect to the query “*What are the total CO*_2_* emissions in different years? Include Scope 1, Scope 2, and Scope 3 emissions if available*.”. Relevance is computed based on cosine similarity between the query embedding vector and the page embedding vector of each page of the report. The pipeline identifies the three most relevant pages in terms of our relevance score and adds the preceding and subsequent page for each of the pages to our pages set. For the data extraction, we prompt the LLM GPT-4 passing the page text of our pages set as context. The prompt queries Scope 1, 2 (market-based), 2 (location-based) and 3 emissions with their respective units for the years 2013-2022 (see Fig. 4 for the full prompt). The LLM returns a string from which the emissions data is extracted using regular expressions and saved to a dataframe where each row contains emissions data, raw LLM output as well as the relevance score of the page referenced, its text and the set of searched pages. The emissions data is identified by a year-scope-page combination.

### Annotation Convention

In a collaborative effort of methodological and domain experts we develop an annotation convention for an emission value (and unit) to be of interest for substantive analysis, that can be applied unequivocally and allows for comparisons between reports, companies and annotators. This annotation rule states that an emission value should be extracted and labeled as correct if it

1. covers emissions for the entire company,
2. is reported according to the operational boundaries of the scopes
3. is reported in absolute terms as *CO*_2_ or *CO*_2_ equivalents emissions and
4. represents a total value, not subcategories.

To exemplify the criteria, we present a positive and a (fictitious) negative text example in Table 1.

**Table 1:** A positive example of a text passage fulfilling all of the criteria for valid value and unit as well as a negative example failing to fulfill any of the necessary criteria.

*Text passage*	*Positive example*
The Scope 1 total emissions according to GHGP for the Allianz Group are reported as 42 kt *CO*_2_e for the year 2019.	*✓* covers emissions for the entire company *✓* is reported according to operational boundaries of the scopes *✓* is reported in absolute terms *✓* represents a total value

*Text passage*	*Negative example*
The GHG emissions in our South Asian facilities were reduced by 2% in 2018 compared to the previous year.	✗ covers emissions for the entire company ✗ is reported according to operational boundaries of the scopes ✗ is reported in absolute terms ✗ represents a total value

There are precise rules describing special cases for each case, which we detail in the guidelines available on Zenodo. This strict convention resulting in a potentially large amount of LLM extractions classified as incorrect prioritizes quality of extractions over quantity and guarantees that the retained entries represent values relevant for climate research, the ground truth. Emission values and units that do not fulfill all four criteria are of no substantive interest and should not be extracted from the PDF and, if extracted by the LLM, annotated as incorrect.

### Non-expert Annotations

In the first annotation phase, seven non-expert annotators annotate the 139 sustainability reports in our sample. We engage annotators with no prior knowledge of non-financial corporate disclosures or environmental accounting, but with a connection to research. Two of them are assigned to each report and the respective LLM annotations. They independently adjudicate the extracted information by the LLM as well as the respective sustainability reports, extract any additional values they find accurate (even if not extracted by the LLM), and mark and describe complexities that occur during this process.

**Fig. 5:**
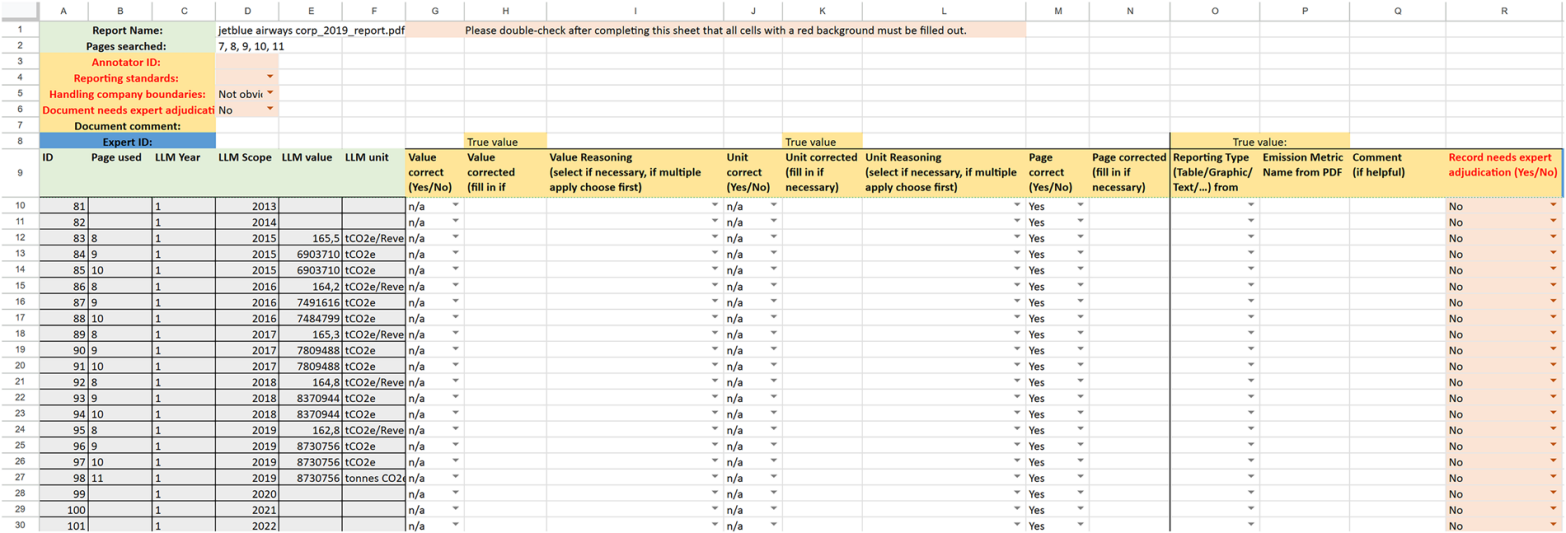
Example annotation spreadsheet.

Extracting the GHG emissions from company reports requires a high degree of domain-specific expertise. To allow for informed non-expert annotations in our initial human annotation phase, a variety of measures are taken. Non-expert annotators are trained in two tutorial meetings where the annotation convention, described above, is extensively explained, examples and common errors are discussed. Between the first and second tutorial meeting, non-experts also perform test annotations which are evaluated and discussed in the second training, where annotators have the opportunity to ask clarifying questions. Annotations are then put into standardized spreadsheet templates (see Fig. 5 for an example). Ultimately, we conduct automated robustness and sanity checks on the provided annotations and instruct the non-experts to correct obvious mistakes such as missing data.

To annotate the extracted information, the non-experts simply check a box whether the LLM output is accurate or not. Additionally, for every incorrect extraction the human annotators select a reasoning from a pre-defined list and specify the corrected value and/or unit. The list of seven error reasonings, which is ordered by priority of errors, can be found in the provided codebook. Allowing for a categorization of LLM errors, this data is a valuable byproduct of the annotation process which can be used by engineers to calibrate prompts in the LLM-based pipeline. Furthermore, information about the page containing the correct value, the display type (e.g., text, table or graphic), and the name of the reporting metric (e.g., “Total Scope 1 emissions”) is collected. Ultimately, the non-expert annotators have the option and are encouraged in the tutorials to request expert adjudications in case of unclear records. This ambiguity marker can be checked for a single scope-year combination (e.g., Scope 2 emissions for 2018) or a whole sustainability report. The non-expert annotators are also instructed to record all mentions of the relevant emissions, which occasionally requires adding rows to the annotation document.

We define two criteria which determine whether a report is routed to experts: If the two non-experts disagree in their assessment of an extracted value or unit for at least one row in a report, the entire report is selected for expert annotation and the respective rows are highlighted for the experts. In addition, the ambiguity marker requesting expert review on row or document level signals that the report is passed on to the experts. This filtering results in 56 reports being passed on to the experts and 83 reports marked as not required to be expert adjudicated.

### Expert Annotations

The study uses expert annotators who specialize in environmental accounting, economics or finance all with thorough knowledge of corporate emission disclosure practices, particularly through the GHGP framework. For each of the reports passed on, our group of four domain experts splits up into two random groups of two, ensuring that all possible pairings occur in a balanced manner, to independently discuss the non-expert disagreements and ambiguities. All of our experts are project team members, just like the non-experts; however, in contrast, they have a background in sustainable finance and have already worked on company report annotation. Being able to discuss challenges with a partner is also expected to improve group decisions. The expert groups are provided with the complete information, i.e., the PDF and the annotations by the LLM and both non-expert annotators. They are asked to decide which of both non-expert annotators is correct and, if neither of them is, to provide a correction. The two groups fully agree on all emission value, unit and page annotations for 28 reports. These reports are considered gold standard. For the 28 remaining reports the two expert groups disagree on at least one scope-year level annotation, and the reports are passed on to the expert discussion.

### Expert Discussion

The adjudication differences between the two expert groups are finally resolved in an in-person discussion of all four experts. The experts are asked to exchange their views, elaborate on written comments and eventually resolve their disagreement. In case the expert group cannot reach an agreement, an entry can be marked as unresolvable. However, this scenario does not occur. The discussion is based on a template containing all previous annotations: LLM, non-expert and expert group annotations. Rows with disagreement between expert groups are highlighted and values resulting from the discussion noted down.

With this multi-staged annotation process we create a high-quality gold standard dataset, that even corrects for disagreements between groups of experts. At the same time the annotations by the LLM and the non-experts reduce the total demand and burden for the group of experts.

## Data Records

All data are accessible through the Zenodo repository^16^. The repository additionally contains the codebook and the detailed annotation guidelines. The data collection, extraction and annotation process described above results in two datasets, a gold standard containing the ground truth data for our sample, and an annotation dataset documenting the results of the sequential steps of the annotation process.

### Gold standard

The gold standard is composed of 5646 data records. Each data record is attributed to one report from the sample, identified by a company name and a reporting year. One record describes a year-scope combination with a value and a unit for that specific report, e.g., the Blackberry report from 2022 lists Scope 1 emissions in year 2019 as 6748 (value) metric tons *CO*_2_*e* (unit). We prompt the LLM to extract emission metrics for 4 different scopes for a year range of 10 years for 139 reports. So we expect 5560 data records. The additional records are due to the fact that the LLM sometimes misses entries and non-experts add them manually during the annotation process. Furthermore, in some cases values beyond the queried year range or multiple mentions of the same scope-year combinations are available in the reports and extracted by the LLM. If the same scope-year combination appears twice in the report on different pages, the page number identifies the specific data record. We also provide the text of the respective page in the dataset as additional source information. About half of the sustainability reports (69 of 139) do not contain any GHG emission values, partly as a consequence of our strict annotation rules.

Among the remaining 70 reports the average number of values reported is 9.2, with two reports containing a maximum of 32 scope-year combinations. As explained in Fig. 2, we extract emissions for four types of scopes. The most frequently reported emission type, as depicted in Table 2, is Scope 1 where an average report features 3.3 mentions. For Scope 2 there is a large difference between market-based and location-based reporting: on average a report contains 2.9 references to location-based Scope 2 emissions while only 1.2 instances of market-based Scope 2 emissions. This is due to our annotation convention which requires to attribute non-explicit mentions to location-based instead of market-based Scope 2. The convention is defined in line with the GHGP, where location-based reporting is described as the default. 1.7 mentions in an average report pertain to Scope 3. The year range used for prompting spans from 2013 to 2022, however 76.4% of the values lie within the range from 2017 to 2021 which is in line with the range chosen in the report collection phase (2017–2022). For each non-missing value and unit combination we also provide a normalized unit which maps a ground truth unit to a unit from the custom normalization dictionary. This dictionary is based on the analysis of a preliminary units set and resolves inconsistencies like different number of spaces in the entry or long and abbreviated units. The metric name and display type extracted manually by the non-experts represent additional record-level information. 80% of all emission values are found in tables in the reports. Roughly 10% of the values are reported in graphics and almost 4% directly in the text. For the remaining 6% of emissions data, the display type is unclear, meaning the non-experts disagree on the display type for these records. In this case, the gold standard dataset features both annotated reporting types, separated by a delimiter.

**Table 2:** Mean number of reported emission values per report by Scope type.

Scope	Average reportings per report
1	3.33
2lb	2.94
2mb	1.23
3	1.71

Furthermore, we present report-level information such as the URL to download the report, and the report type. 51 reports are annual reports. The remaining 88 reports differ highly in the naming of their sustainability report: 49 are titled sustainability reports, 18 are referred to as ESG reports and the rest are several variations of the terms “sustainability” and “responsibility”.

### Annotation dataset

To allow for an in-depth analysis of the annotation quality and behavior, we provide an additional dataset documenting the intermediate steps of the annotation process which we denote by annotation dataset. A first block of columns refers to the data extracted by the LLM, the other columns can be grouped into columns annotated by non-experts and experts. The ordering of columns mimics the structure of the annotation spreadsheet templates we provide on GitHub. This dataset can be used, for example, to evaluate the importance of expert annotation by analyzing agreement between non-expert and experts. In 97% of cases, experts agree with the value annotated by one of the non-experts. Only in roughly 3% of the cases they set a value different from the non-expert annotations. In addition, the annotated reasons for LLM errors can serve to better understand the underlying causes of these errors, guiding improvements in prompt design and model training.

## Technical Validation

We implement several data checks for the different steps of the annotation process. Taking the LLM extractions as given, we analyze the non-expert annotations for different criteria. Missing assessments of value or unit for records where the LLM extracted some values signal missed annotation and the report is resent to the non-expert. The same applies to missing annotator ID which is again a compulsory entry in the annotation process. Another check involves the number of rows: we list the number of rows per report and inspect outliers manually. Annotators sometimes miss to enter the assessment, but correct the value (analogously for unit and page). These kinds of discrepancies are resolved automatically. To distinguish LLM-generated records from manually added we create an ID for the added records.

Report pass-over to the experts is determined by agreement of the two non-experts on the assessment of the value and unit, i.e., whether both annotators entered “Yes” or “No” in the column “Value correct” (and analogously for unit). If “No” (not correct), non-experts are asked to enter a corrected value. When a report is passed on to experts, however, only the assessment is considered, not the corrected value. In case of differences in the corrected value we manually inspect the records in order to decide on a ground truth value.

Similar checks are also implemented when joining the expert annotations with the already existing data. Annotation mistakes with respect to the annotation convention also arise among experts and are resolved in the expert discussion round.

Ultimately, we inspect the gold standard dataset for logical inconsistencies to ensure correct data generation and processing.

## Usage Notes

### Data and Scripts

The scripts, written in R and accessible through GitHub, ensure reproducibility of the study’s results. Every step, from raw annotated data to the processed dataset, is described in the scripts. Two key points are particularly noteworthy: the normalization convention for emission units is detailed in the provided script unit_normalization.Rmd. When loading data files, users should load the page column as characters due to non-numeric page names in the PDFs (e.g., “Env33”). Users can match the two datasets (gold standard and annotation dataset) using the variable combination of company_name, report_year and merge_id. The merge_id already includes the company name and report year implicitly, but to avoid column duplication in the join operation, it should be included as join variables. Furthermore, the gold standard also provides the URLs to access the sustainability reports.

### Reuse Potential

The datasets inherit large re-use potential due to the gold standard nature of the emission metrics and the accompanying wealth of information. The two-stage expert adjudication process ensures high quality, making the dataset a benchmark for human and automated annotation/information extraction systems. For automated approaches, the dataset facilitates the development, engineering, and alignment of LLM prompts to replicate its contents. The additional metadata, such as the page location of emission values, verbatim descriptions of the metrics, and display types, enhance the dataset’s methodological value. This information aids in understanding the strengths and weaknesses of current automated extraction methods. The annotation dataset also documents a scalable data collection pipeline combining non-expert annotators with targeted expert input, offering a model for future data collection efforts. This effort serves as a starting point for a potential upscaling of extracting emission information from sustainability reports. For example, the gold standard data could be re-used to fine-tune a synthetic expert and extend the extraction task to a broader set of sustainability reports. Similarly and moving beyond corporate disclosures, countries have to report their national emissions according to the Paris Agreement in Biennial Update Reports and Nationally Determined Contributions, that is, in PDF files. The data collection pipeline we describe here could be adopted to extract data from these country reports, among others. Our data can support substantive analyses from a sustainable finance standpoint, attempting to uncover patterns around scope emission reporting. Researchers could identify factors that are correlated to how companies report their emissions such as business domain, region or reporting year by combining our dataset with other datasets. These analyses can also inform both report authors and those designing the surrounding governance frameworks on how reporting practices should be structured to support automated information extraction, thereby contributing to greater transparency and accountability.

### Limitations

Generalizability of our sample of data is limited by a relatively small number of reports. Additionally, the assumption that agreement between two non-experts guarantees gold standard quality introduces potential bias. Data users need to be aware of this annotation convention and, if in doubt, limit their analysis to reports that underwent expert adjudication review. Generally, all reported Scope 3 emissions need to be treated with caution, as their optional and therefore often incomplete reporting makes comparisons between companies challenging. We try to address this comparability issue by only including “Total Scope 3” emissions in the dataset rather than single subcategories. However, since the definition of what can be considered total rests with the reporting entity, a degree of uncertainty remains. Relatedly, all GHG emissions reported by companies in accordance with the GHGP are subject to uncertainties as these are mostly calculated based on activity measures and emission factors [^9^, p.11] rather than directly measured and subject to assumptions and choices made by the reporting entity^17^. Ultimately, it is unclear to what degree human annotators are subject to confirmation bias, leading to a tendency to confirm the LLMs extraction or the decision of a preceding annotator.

## Data Availability

All code files can be accessed via the GitHub repository https://github.com/soda-lmu/gist-data-descriptor.
